# Modeling vehicle collision risk for the jungle cat in the Hyrcanian forests of Iran: A guide for vehicle collision prevention

**DOI:** 10.1371/journal.pone.0336611

**Published:** 2026-05-14

**Authors:** Abbas Ashoori, Anooshe Kafash, Koros Rabiei, Mojtaba Hosseini, Shapour Abdi, Farhad Hosseini Tayefeh, Sayedeh Alemohammad, Masoud Yousefi

**Affiliations:** 1 Gilan Provincial Office, Department of the Environment, Rasht, Iran; 2 School of Culture and Society, Aarhus University, Aarhus, Denmark; 3 Mazandaran Provincial Office, Department of the Environment, Sari, Iran; 4 Golestan Provincial Office, Department of the Environment, Gorgan, Iran; 5 Gilan Provincial Office, Department of the Environment, Amlash, Iran; 6 Research Group of Biodiversity and Biosafety, Research Center for Environment and Sustainable Development (RCESD), Department of Environment, Tehran, Iran; 7 Faculty of Governance, University of Tehran, Iran; 8 Department of Animal Science, School of Biology, Damghan University, Damghan, Iran; 9 LIB, Museum Koenig, Bonn, Leibniz Institute for the Analysis of Biodiversity Change, Bonn, Germany; Salim Ali Centre for Ornithology and Natural History, INDIA

## Abstract

Wildlife-vehicle collisions are an important wildlife conservation challenge, especially for carnivores. In Iran, such vehicle collisions pose a major threat to carnivores. The jungle cat (*Felis chaus*) is a small carnivore species facing multiple threats, including habitat destruction, land use changes, and particularly vehicle collisions. We collected data on jungle cat vehicle collisions in the Hyrcanian forests of northern Iran to model the jungle cat vehicle collision risk and identified high vehicle collision risk areas within the 1 km and 5 km buffers. To check validity of Maxent results, we additionally carried out a binomial Generalized Linear Model (GLM). Results showed that western Golestan province, eastern Mazandaran province, and central Gilan province as the highest vehicle collision risk for the jungle cat in the Hyrcanian forests. According to the Maxent model, human footprint and slope and based on the GLM model, NDVI and human footprint were the most important predictor of jungle cat vehicle collision risk in the Hyrcanian forests. We recommend to implement roadside vegetation management and wildlife crossings in the high collision-risk areas in the Hyrcanian forests to mitigate road mortality and support conservation planning for jungle cats in Iran.

## 1 Introduction

Human development including the constructions of roads, pose serious threat to biodiversity worldwide [1-9]. Mobile species, particularly carnivores, are vulnerable to the impacts of road development in human-modified landscapes [10,11]. Roads may substantially diminish carnivore populations and distributions by increasing mortality as a result of vehicle collisions, creating barriers to movement, altering animal behaviours and habitat use patterns, degrading habitats through changes in the physical and chemical conditions, noise pollution, and decreasing the quantity and habitat fragmentation [1,12,5,13,14]. Increased road density also facilitates human access, which can disturb wildlife [15].

In response to these threats, conservation biologists are evaluating the impacts of roads on biodiversity and developing strategies to reduce their negative impacts [1,6,8]. For example, recent recognition of the substantial impacts of roads on large mammal populations has prompted many efforts to design and realign roads to reduce road mortality [4,16-19]. Most research on road impacts and mitigation has been conducted in developed countries [20,4,16,21,5,6,22,23,17,24], where road collisions are well documented [20,25]. For instance, 51,522 animal collisions were recorded in Texas, from 2010 to 2016, these resulted in 254 human fatalities and 6,914 injuries, in addition to animal loss [25].

Species Distribution Models (SDMs) are being used to identify areas showing high road collisions probability and environmental predictors of collisions within the defied geographic area [22,17]. Wright et al. (2020) [17] used SDMs to identify areas of high probability of European hedgehog (*Erinaceous europaeus*) roadkill occurrence across the British road network [26]. In another study, Fabrizio et al. (2019) [22] applied SDMs to determine roadkill risk areas for the Eurasian badger (*Meles meles*) in the Abruzzo region (Central Italy) [27]. In contrast, the impacts of roads on biodiversity remained relatively unknown in many developing countries compared to developed countries [28]. For instance, road networks in Iran pose a serious conservation issue for many wildlife species [29,28,30]. Naderi et al. (2018) [30] found that road mortality was the second most frequent cause of unnatural mortality in Persian leopard (*Panthera pardus*) in Iran, with northern Iran showing higher mortality risks. Jungle cat (*Felis chaus*) have also been identified as vulnerable to road mortality in the region [31].

Northern Iran is largely covered by relict deciduous Hyrcanian forests, which comprise a continuous 800 km and an area of over 1.8 million hectares [32-35], support high biological diversity and endemism [32,36-41]. However, this area is rapidly changing due to urbanization and agricultural expansion, resulting in the native fauna becoming vulnerable [42-44]. Hyrcanian forests are home to many endangered and ecologically important mammals including Persian leopard (*Panthera pardus*), Eurasian lynx (*Lynx lynx*), jungle cat (*Felis chaus*) and probably the wild cat (*Felis lybica*) [ 45,46]. Climate and land use changes, grazing, illegal hunting, and road mortality are important anthropogenic causes of biodiversity loss in this region [33,44,30,47,37,48].

Jungle cat mortality rates in the Hyrcanian forests were reported to be higher than other parts of the country, therefore monitoring and mitigation efforts are important. Our goal was to develop a vehicle collision risk model for the jungle cats in the Hyrcanian forests, identify the most influential environmental factors contributing to vehicle collision risk, and map high vehicle collision risk areas within and outside protected areas. We hypothesized that vehicle collision risk is higher in areas with higher human development and road density. We also hypothesized that jungle cat vehicle collision would be higher in areas of dense roadside vegetation cover because of lower sight distance by both driver and cats in such areas.

## 2 Materials and methods

### 2.1 Study area

**Fig 1:**
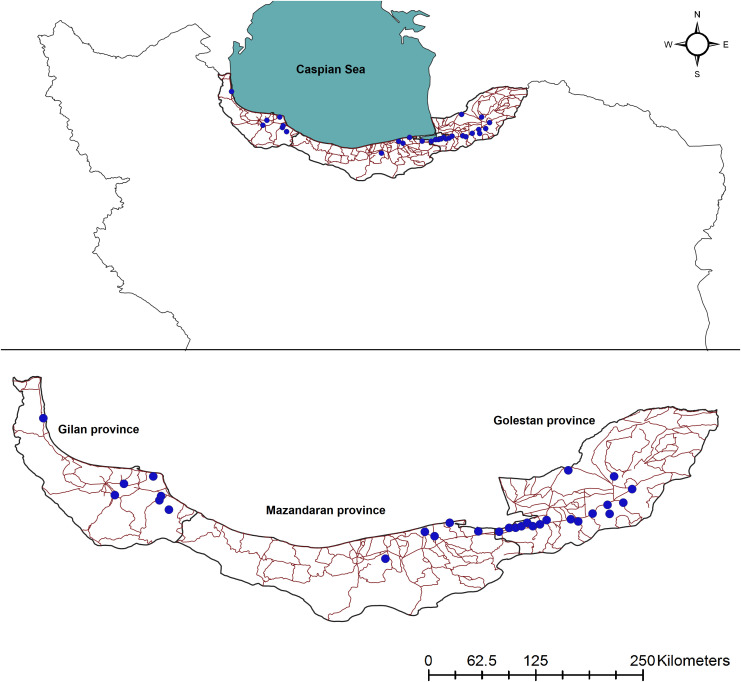
Location of study area in Iran (a). Roads (https://www.openstreetmap.org/) and jungle cat vehicle collision locations **(b)**. The study areas consisted of Hyrcanian forests in three Iranian provinces: Gilan, Mazandaran and Golestan. Maps were created in QGIS 3.44 (https://qgis.org/). This map contains information (roads) from OpenStreetMap and OpenStreetMap Foundation, which is made available under the Open Database License.

The study area consisted of Hyrcanian forests in three Iranian provinces: Gilan, Mazandaran and Golestan (Fig 1). The area extends along the Caspian Sea from the Iran-Azerbijan Republic border on the southwest side to Golestan National Park on the southeast side (Fig 1). Annual precipitation mainly ranges between 530 and 1350 mm, but can reach up to 2000 mm in the west [33]. This makes the area significantly wetter than most other parts of Iran, where average annual precipitation is around 250 mm. The study area has a mild subtropical humid climate that is favorable for agriculture and includes a center of food production for Iran [49]. The landscape is largely rural, characterized by small towns, low-density farmlands, and grazing lands.

### 2.2 Study species

The jungle cat is found in Egypt of North Africa and is widespread in Asia from the Middle East, Southwest Asia through Central and South Asia to Southeast Asia, reaching Indochina and possibly the Malayan Peninsula [50-53]. The species has been reported to occur throughout most of Iran except in deserts [46,54,31]. The species occurs at elevations from −28 m to 4,178 m asl., the widest elevational distribution of the eight living felid species of Iran [46,31]. Despite this wide distribution, the jungle cat is one of the least studied felid species in Iran [46,31]. To date, only one study has examined its distribution, ecology and conservation in Iran [31]. Populationsin Iran has been reported to have decreased significantly over the past years [55]. It is cited in the Appendix II of CITES, listed as “least concern” in the IUCN Red List [53] and is legally protected in Iran [46].

### 2.3 The jungle cat vehicle collision data

Vehicle collisions data were collected opportunistically from March 2016 to April 2019 in northern Iran (Fig 1). We recorded the coordinates of 30 collision sites, when we could confidently confirm the identification of the species using Global Positioning System (GPS). We then checked the occurrence data of collision sites for spatial autocorrelation and applied the global Moran’s test to evaluate the structural pattern of the data.

### 2.4 Environmental data

We used climatic, topographic, anthropogenic, and Normalized Difference Vegetation Index (NDVI), and variables to characterize collision sites and identify the most important predictors of collision risk. Climatic variables were downloaded from the WorldClim database at 30-seconds spatial resolution [56] including: isothermality) Bio3), seasonal temperature change (Bio4), mean temperature of wettest season (Bio8), mean temperature of driest season (Bio9), seasonal precipitation change (Bio15), precipitation of warmest season (Bio18), and precipitation of coldest season (Bio19). We used NDVI as an indicator of primarily productivity and vegetation cover within the study area. We considered the human footprint index [57] as a measure of anthropogenic impact. This index is based on the following factors: 1) built environments, 2) population density, 3) electric infrastructure, 4) crop lands, 5) pasture lands, 6) roads, 7) railways, and 8) navigable waterways [58]. Then to create the standardized human footprint map all eight above mentioned factors were overlaid [58]. Slope and topographic heterogeneity were obtained from the Shuttle Radar Topography Mission (SRTM) elevation model [59]. We calculated variance inflation factor (VIF; 60) for the variables using ‘usdm’ package [61] in R 4.4.3 [62]. The results showed that collinearity was low (VIF < 10) among variables: Bio3 = 3.399, Bio4 = 6.38, Bio8 = 7.917, Bio9 = 8.127, Bio15 = 1.833, Bio18 = 6.42, Bio19 = 3.739, slope = 5.882, topographic heterogeneity = 3.502, NDVI = 1.885 and human footprint = 1.756.

### 2.5 Vehicle collision risk modeling and variable importance

Maximum entropy modeling (Maxent) is the most popular algorithm among many species distribution modeling algorithms [63,64] and it has been widely applied across various scientific disciplines including ecology, biogeography, conservation, evolution, health geography and even archaeology [17,65-70]. In this study, we used Maxent to predict areas with high vehicle collision risk in the study area [71,72]. To run Maxent, we considered the Kuenm R package [73]. The kuenm R package is designed to facilitate more robust, reproducible model calibration and final model development by generating suites of candidate models and optimizing parameter settings for each study [73]. We applied this package to create Maxent candidate models with multiple combinations of regularization multipliers, feature classes, and background points. Then, the best parameters for modeling were selected based on the statistical significance, predictive power, and model complexity [73]. We removed environmental variables with contributions less than 1%. Additionally, we modeled the jungle cat vehicle collision risk in the study area but by creating 1 km and 5 km buffers around the roads and identified high vehicle collision risk areas within the 1 km and 5 km buffers.

The performance of the vehicle collision risk models was assessed using the area under the curve (AUC) metric of the receiving operator characteristic (ROC) curve [71]. AUC is commonly used as a measure of model performance in ecological studies [72,74,75]. AUC ranges from 0 to 1 an AUC value of 0.5 indicates that the performance of the model is not better than random, while values closer to 1 indicate better model performance [76].

In order to check validity of Maxent results, we additionally carried out a binomial Generalized Linear Model (GLM) with a logit link function following Yousefi et al. (2018) [77]. For the 30 vehicle collision sites we obtained 120 random locations across the study area. To reduce collinearity, we used valuables with low variance inflation factor (VIF < 10) that were identified as important (contributions more than 1%) in the Maxent model (Bio3 = 3.399, Bio9 = 8.127, Bio19 = 3.739, slope = 5.882, NDVI = 1.885, human footprint = 1.756). We computed GLM models with all possible variable combinations then applied Akaike’s information criterion adjusted to small data sets (AICc) for model selection [78]. We defined our top concurrent models as those that fell within two AICc (Δ AICc < 2). As for the GLM models, eight concurrent models had a ΔAICc < 2 and were retained. Analyses were performed in R 4.4.3 [62], using the MuMIn R package version 1.48.11 [79].

### 2.6 High vehicle collision risk areas within protected areas

To estimate high vehicle collision risk areas for the jungle cat within protected areas, we first converted the continuous vehicle collision risk map to a binary map using the maximum test sensitivity plus specificity threshold (cut of value 0.21) [71,80]. Then we overlaid the binary map with protected areas of Iran [81] and calculated vehicle collision risk area within protected areas network. The most recent shapefile of Iran’s protected areas was obtained from Department of Environment of Iran.

## 3 Results

### 3.1 Vehicle collision risk model

**Fig 2:**
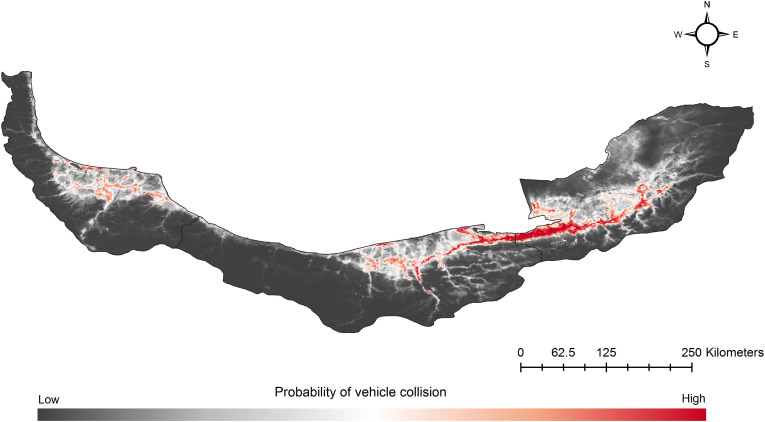
Relative probability of the jungle cat vehicle collisions across the study area. The collision risk model is developed based on 30 jungle cat vehicle collision locations and Maxent model by considering following environmental variables: human footprint, slope, NDVI, distance to wetlands, distance to rivers, isothermality (Bio3), mean temperature of driest season (Bio9) and precipitation of coldest season (Bio19) at 30-seconds spatial resolution. Map was created in QGIS 3.44 (https://qgis.org/).

We recorded 30 jungle cat mortality incidents from the three northern Iran provinces. All 30 observations were directly recorded by the authors or their colleagues, and jungle cat carcasses were observed. However, due to delays in discovery, often occurring days after the incidents it was not possible to determine their sex or collect biometric data. Mortality incidents occurred at elevations between 0 m and 437 m above sea level, with an average elevation of 68 m across the 30 recorded cases. All incidents took place on primary asphalt roads. Based on the AUC metric of the ROC curve, the overall predictability of the jungle cat vehicle collisions model was high (AUC = 0.906). The Maxent model showed that the highest risk of vehicle collisions for the jungle cat in the study area occurred within western and central Golestan, eastern Mazandaran and central Gilan (Fig 2).

### 3.2 Road based vehicle collision risk models

**Fig 3:**
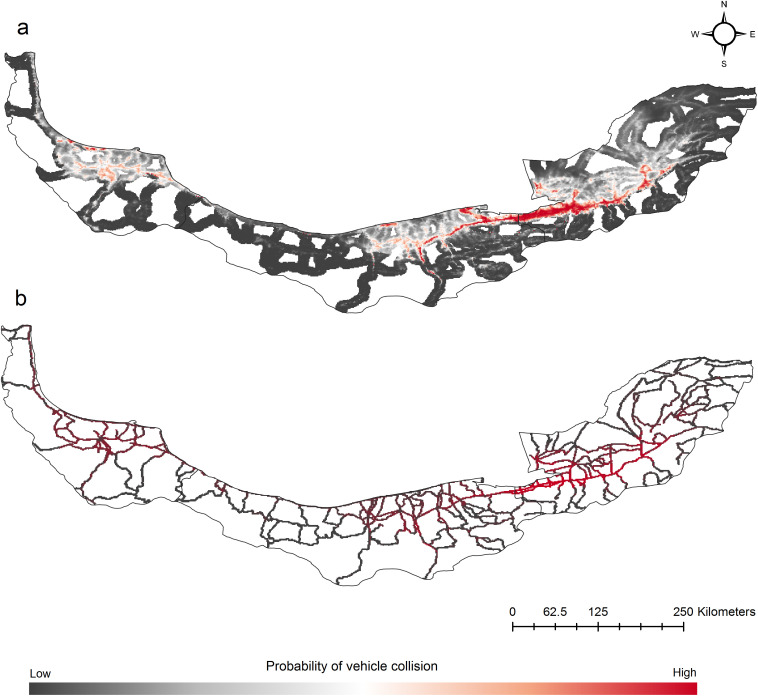
Relative probability of the jungle cat vehicle collisions based on Maxent model based on the 5 km (a) and 1 km (b) buffers around the roads. The models were created by considering following environmental variables: human footprint, slope, NDVI, distance to wetlands, distance to rivers, isothermality (Bio3), mean temperature of driest season (Bio9) and precipitation of coldest season (Bio19) at 30-seconds spatial resolution. Maps were created in QGIS 3.44 (https://qgis.org/).

We predicted the jungle cat vehicle collision risk areas by creating 1 km and 5 km buffers around the roads. We found that roads crossing western and central Golestan, eastern Mazandaran and central Gilan were associated with highest vehicle collision risk (Fig 3). Based on the AUC metric of the ROC curve, the overall predictability of the jungle cat vehicle collisions models at 5 km buffer (AUC = 0.841) and 1 km buffer (AUC = 0.883) was high.

### 3.3 Variable contribution

Human footprint and slope were the most important predictor of the jungle cat vehicle collision, with 48.3% and 17.2% contributions respectively (Table 1). Human footprint and jungle cat vehicle collision risk were positively correlated. Slope and jungle cat vehicle collision risk were negatively correlated, with lower incidence of collisions in steeper areas. The probability of jungle cat vehicle collisions also increased with an increase in NDVI values while the probability decreased with an increase in distance from wetlands and rivers. Results of variable importance were similar when modeling area was limited to a 5 km buffer zone around the roads. But when modeling area was limited to a 1 km buffer zone around the roads, slope which was the second most important predictor became insignificant however, human footprint was still the most important predictor of the jungle cat vehicle collision.

**Table 1:** Contributions (%) of environmental variables to the vehicle collision risk model for the jungle cat in the Hyrcanian forests, northern Iranian provinces.

Variable	Contribution in first step	Contribution in second step	5 km buffer	1 km buffer
Human footprint	46.6	48.3	48	49.1
Slope	16.6	17.2	19.4	2.5
Mean temperature of driest season (Bio9)	15.4	14.5	14.7	29.2
Normalized Difference Vegetation Index (NDVI)	6.7	6.6	3.3	9.5
Distance to wetlands	6.0	6.4	6.1	1.3
Precipitation of coldest season (Bio19)	3.6	4.0	6	5
Distance to rivers	1.4	1.6	1.3	1.1
Isothermality (Bio3)	1.3	1.4	1.2	2.2

Eight retained models and AICc and ΔAICc values were presented in Table 2. Results of model averaging then showed that NDVI (with the maximum importance of 1) and human footprint (with the maximum importance of 0.95) were the most important predictors of the jungle cat vehicle collisions (Table 3). Both variables positively correlated with the incidence of vehicle collisions (Table 3). The GLM and model averaging procedure therefore confirmed the Maxent analysis, that human footprint and NDVI were the most important predictor in the variables we considered.

**Table 2:** AICc results for the combination of all models, also indicating ΔAICc.

Models	NDVI	Human footprint	Bio9	Slope	Distance to wetlands	Distance to rivers	Bio3	Bio19	df	logLik	AICc	ΔAICc
Model 1	×	×	×	×	×				6	−13.22	39.04	0.00
Model 2	×	×	×	×	×	×		×	7	−12.22	39.24	0.20
Model 3	×	×	×		×				5	−14.52	39.46	0.42
Model 4	×	×	×		×				5	−14.79	40.00	0.96
Model 5	×	×	×	×	×		×		7	−12.84	40.48	1.44
Model 6	×	×	×	×		×			6	−14.01	40.62	1.58
Model 7	×	×	×	×	×	×	×		8	−11.81	40.65	1.61
Model 8	×	×	×		×	×			6	−14.08	40.75	1.71

**Table 3:** Coefficients of the model averaging procedure, indicating the relative importance of the variables.

	Coefficients	Variable importance
Intercept	−3.469e + 01	---
NDVI	1.220e-01	1
Human footprint	2.199e-01	0.95
Bio9	8.008e-02	0.86
Slope	−1.686e-01	0.8
Distance to wetlands	−3.744e + 00	0.66
Distance to rivers	−2.298e + 01	0.45
Bio3	7.254e-03	0.36
Bio19	6.881e-04	0.27

### 3.4 Protected areas coverage

**Fig 4:**
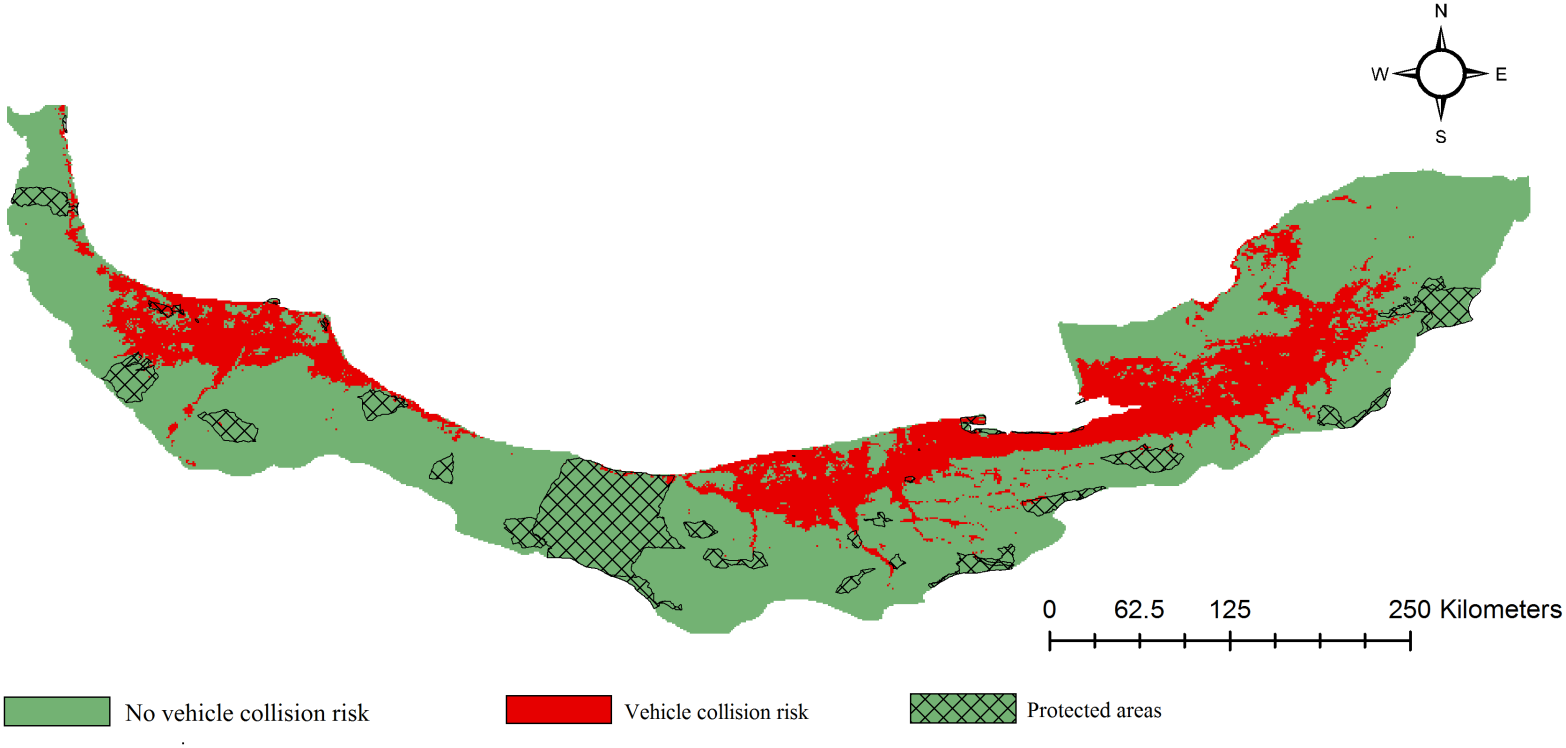
Relative risk of vehicles for the jungle cat in relation to protected areas. Map was created in QGIS 3.44 (https://qgis.org/).

We calculated the extent to which areas of high vehicle collision risk for the jungle cat occurred within protected areas. Results showed that 13,878 km^2^ of the study areas have high vehicle collision risk for the species and only 213 km^2^ (1.5 percent) of the high risk areas were located within protected areas (Fig 4).

## 4 Discussion

Road development is a serious threat to carnivore species [82] because of their large home ranges and need to maintain population. Therefore, understanding the measures of this phenomenon is vital to find spatial distribution of roadkill hotspots and to mitigate its effect on carnivore population [83]. Our quantification of environmental characteristics associated with jungle cat vehicle collisions risk found that human footprint, slope and NDVI were the main predictors of vehicle collision risk in the Hyrcanian forests of Iran.

Human footprint variable was the most important determinant of the jungle cat vehicle collision risk based on the Maxent model and the second most influential according to GLM model and, as expected, when extent of the human footprint increased the probability of vehicle collision also increased. The relationship between human footprint and jungle cat mortality is somewhat surprising, in that areas of highest human footprint would be expected to be too degraded to provide suitable or high value habitat for the species. The species avoids areas with the highest human density especially highly urbanized areas, but it utilizes the outskirts of urban areas, and readily penetrates smaller cities and villages which are the most typical form of human development in the study area. In fact, the cat appears to favor small urban areas and villages because of the augmented availability of prey such as domestic chickens (*Gallus gallus*). This use pattern demonstrates the species’ tolerance of some degree of habitat modification [52], as shown by records of breeding within villages (A. Ashoori, pers. obs.).

NDVI was identified as the most influential predictor of the jungle cat vehicle collision risk based on the GLM model with a positive association. Plant cover plays a key role to the life of jungle cat, which favoured woodlands and shrublands more than other land cover types [31]. Our results, which were confirmed by both Maxent and GLM models, demonstrate that plant cover adjacent to roads could increases the frequency of roadkill. Therefore, keeping lower plant cover around roads could probably reduce the rate of roadkill. Similarly, the rate of roadkill of European hedgehog (*Erinaceous europaeus*) increased with the extent of grassland cover [17]. Therefore, conserving open forests along roadsides, which could provide cover favored by the jungle cat but also increase visibility among cats and vehicles is recommended [52]. Removal of plant cover adjacent to roads, however, may also increase the barrier effect of roads to jungle cat movement. More research is needed to understand how roads and vegetation clearing along roads may fragment habitats and affect road mortality.

According to the Maxent model, slope was the second most important predictor of jungle cat vehicle collision risk in the Hyrcanian forests. However, it did not rank among the top two predictors in the GLM model. The relationship showing that mortalities primarily occur on low slopes could be interpreted to suggest that roads are more likely to occur on flatter lands, rather than reflecting the response of the species to slope [46]. The complete absence of road kill reports from mountainous areas, as well as a previous study [84], both indicate that the jungle cat avoids steep slopes and higher elevation areas [46]. Slope was also the second most important determinant of the species vehicle collision risk when modeling areas was limited to a 5 km buffer zone around roads but it did not come out to be important when modeling areas was limited to a 1 km buffer zone around the roads.

We acknowledge certain limitations in interpreting and applying our study results. Although it may be obvious, it is important to stress that vehicle mortality records represent the intersection of the distributions of the jungle cat and road network. The data do not represent an unbiased estimate of the range or abundance of the jungle cat in any particular geographic region, but only the range and abundance of vehicle collision mortality within sampled areas. Therefore, caution is required in interpreting relationships between road mortalities and certain variables, as relationships may more reflect conditions where roads occur rather than where jungle cats occur. For example, the strong relationship between human footprint and cat mortalities may not reveal anything about the species’ population response to development. Rather, the result may just indicate that the presence of more humans, roads, and vehicles result in higher mortality regardless of the density of species.

Our results suggest several worthwhile areas for further research. Determining the density of roads in suitable habitat could be helpful in further determining what proportion the species range is subject to roadkill. Quantifying the number of kills/road mile/year could also improve the characterization of potential population impacts. Developing a systematic reporting system for road mortalities by enlisting highway maintenance workers and interested community members, would provide a better indication of the frequency of roadkills, areas of the species range with higher and lower roadkill rates, and roadside conditions associated with vehicle mortalities. Further information derived from roadkill hotspots should be helpful in mitigating when construction of new roads (such as locating road alignments away from riparian and wetland habitats) but offers fewer options for existing roads (i.e., warning signs, installing lighting, vegetation clearing, enhancement of existing undercrossings to facilitate movements). Future studies are needed to more finely quantify conditions at the immediate site of impact (i.e., number of road lanes, traffic volume, and multi-lane roads) as well as jungle cat density in areas where roadkills occur. Studies of the effectiveness of measures to reduce mortalities are also needed. For example, vegetation clearing and installation of lighting at areas of high roadkill risk may reduce mortalities, but it also may prevent or reduce jungle cats from using these areas, potentially resulting in population fragmentation. Our mapping of mortalities at existing roads may partially reflect conditions created by impacts of previous roadkill [85].

Results of our evaluation of the overlap of collision occurrence and risk and protected areas deserve careful interpretation. The location of all collision incidents and most areas of high collision risk outside of protected areas demonstrates that collision risk is not high in these areas, but we only have limited knowledge that suggests that these areas, based on their location in more montane topography, may support lower densities of jungle cat than unprotected lowlands. If protected areas do not support high jungle cat densities, additional land protection may be warranted to protect the species in areas where it is now at risk from vehicle collisions.

It should be noted that in addition to the jungle cat, several other ecologically significant mammal species such as the brown bear, Persian leopard, Eurasian lynx, and potentially the wild cat face high vehicle collision risk in the Hyrcanian forests [86-88]. We believe the findings of this study may also be valuable for these species, as areas with dense vegetation and increased human footprint near asphalt roads pose elevated collision risks for various mammalian fauna. Therefore, keeping lower plant cover around roads could probably reduce vehicle collision risk for other mammal species in the study area.

## 5 Conclusion

In this study we showed that areas with a high human footprint and vegetation and low slope around road network in Hyrcanian forests have high vehicle collision risk for the jungle cat. Almost all high vehicle collision risk areas are outside protected areas, but the presence of many protected lands in areas of high slope [81] makes it uncertain to what degree these protected areas support jungle cat populations. Using roadside fences to direct jungle cat movements to areas below roads (under bridges or through culvets) could be effective in reducing road crossings and resulting vehicle collisions. We also recommended experimental treatments to reduce vegetation cover in zone from ground level to 2 m by tree thinning or removal of shrub and herbaceous vegetation beside roads near river crossings and wetlands, where jungle cats are most likely to occur. Partial overhead tree cover should be retained in these areas because the species appears to prefer such areas and the presence of tree cover also will discourage the growth of low vegetation and thereby retain desirable conditions and reduce the frequency of vegetation treatment. As roads appear to be a threat for the jungle cat, we recommend quantifying vehicle collision risk across the species distribution range in Iran to be able to apply measures to reduce road mortality. We also recommend quantification of vehicle collision risk for other ecologically significant mammal species such as the brown bear, Persian leopard and Eurasian lynx in the study area [89].

## References

[1] Forman RTT, Sperling D, Bissonette JA, Clevenger AP, Cutshall CD, Dale VH. Road ecology: science and solutions. Washington, DC: Island Press; 2003.

[2] Coffin AW. From roadkill to road ecology: a review of the ecological effects of roads. Journal of Transport Geography. 2007;15(5):396–406. doi: 10.1016/j.jtrangeo.2006.11.006

[3] Laurance WF, Goosem M, Laurance SGW. Impacts of roads and linear clearings on tropical forests. Trend Ecol Evol. 2009;24:659–69. 10.1016/j.tree.2009.06.009 19748151

[4] Gunson KE, Mountrakis G, Quackenbush LJ. Spatial wildlife-vehicle collision models: a review of current work and its application to transportation mitigation projects. J Environ Manage. 2011;92(4):1074–82. doi: 10.1016/j.jenvman.2010.11.027 21190788

[5] Laurance WF, Clements GR, Sloan S, O’Connell CS, Mueller ND, Goosem M, et al. A global strategy for road building. Nature. 2014;513(7517):229–32. doi: 10.1038/nature13717 25162528

[6] Ward AI, Dendy J, Cowan DP. Mitigating impacts of roads on wildlife: an agenda for the conservation of priority European protected species in Great Britain. Eur J Wildl Res. 2015;61(2):199–211. doi: 10.1007/s10344-015-0901-0

[7] Baxter-Gilbert JH, Riley JL, Neufeld CJH, Litzgus JD, Lesbarr`eres D. Road mortality potentially responsible for billions of pollinating insect deaths annually. J Insect Conserv. 2015;19:1029–1035.

[8] Bennett VJ. Effects of road density and pattern on the conservation of species and biodiversity. Curr Landscape Ecol Rep. 2017;2(1):1–11. doi: 10.1007/s40823-017-0020-6

[9] Medrano‐Vizcaíno P, Grilo C, Silva Pinto FA, Carvalho WD, Melinski RD, Schultz ED, et al. Roadkill patterns in Latin American birds and mammals. Global Ecol Biogeogr. 2022;31(9):1756–83. doi: 10.1111/geb.13557

[10] Packer C, Loveridge A, Canney S, Caro T, Garnett ST, Pfeifer M, et al. Conserving large carnivores: dollars and fence. Ecol Lett. 2013;16(5):635–41. doi: 10.1111/ele.12091 23461543

[11] Ripple WJ, Estes JA, Beschta RL, Wilmers CC, Ritchie EG, Hebblewhite M, et al. Status and ecological effects of the world’s largest carnivores. Science. 2014;343(6167):1241484. doi: 10.1126/science.1241484 24408439

[12] Jaeger JAG, Fahrig L. Effects of road fencing on population persistence. Conserv Biol. 2004;18(6):1651–7. doi: 10.1111/j.1523-1739.2004.00304.x

[13] Trombulak SC, Frissell CA. Review of ecological effects of roads on terrestrial and aquatic communities. Conserv Biol. 2000;14(1):18–30. doi: 10.1046/j.1523-1739.2000.99084.x

[14] Vale CG, Tarroso P, Brito JC. Predicting species distribution at range margins: testing the effects of study area extent, resolution and threshold selection in the Sahara–Sahel transition zone. Div Distribut. 2013;20(1):20–33. doi: 10.1111/ddi.12115

[15] Iuell B. Wildlife and traffic: a European handbook for identifying Conflicts and designing solutions. Utrecht, The Netherlands: KNNV Publishers. 2003.

[16] Neumann W, Ericsson G, Dettki H, Bunnefeld N, Keuler NS, Helmers DP, et al. Difference in spatiotemporal patterns of wildlife road-crossings and wildlife-vehicle collisions. Biological Conservation. 2012;145(1):70–8. doi: 10.1016/j.biocon.2011.10.011

[17] Wright PGR, Coomber FG, Bellamy CC, Perkins SE, Mathews F. Predicting hedgehog mortality risks on British roads using habitat suitability modelling. PeerJ. 2020;7:e8154. doi: 10.7717/peerj.8154 31998548 PMC6979406

[18] Vilela T, Malky Harb A, Bruner A, Laísa da Silva Arruda V, Ribeiro V, Auxiliadora Costa Alencar A, et al. A better Amazon road network for people and the environment. Proc Natl Acad Sci U S A. 2020;117(13):7095–102. doi: 10.1073/pnas.1910853117 32179680 PMC7132287

[19] Silva I, Crane M, Savini T. The road less traveled: addressing reproducibility and conservation priorities of wildlife-vehicle collision studies in tropical and subtropical regions. Global Ecology and Conservation, 2021;27:1– 12.

[20] Langbein J, Putman R, Pokorny B. Traffic collisions involving deer and other ungulates in Europe and available measures for mitigation. In: Putman R, Apollonio M, Andersen R, editors. Ungulate management in Europe: problems and practices. Cambridge; 2011.

[21] Marcantonio M, Rocchini D, Geri F, Bacaro G, Amici V. Biodiversity, roads, landscape fragmentation: two Mediterranean cases. Appl Geo. 2013;42:63–72.

[22] Fabrizio M, Di Febbraro M, D’Amico M, Frate L, Roscioni F, Loy A. Habitat suitability vs landscape connectivity determining roadkill risk at a regional scale: a case study on European badger (Meles meles). Eur J Wildl Res. 2019;65(1). doi: 10.1007/s10344-018-1241-7

[23] Loraamm RW, Downs JA, Lamb D. A time‐geographic approach to quantifying wildlife–road interactions. Transactions in GIS. 2018;23(1):70–86. doi: 10.1111/tgis.12497

[24] Grilo C, Koroleva E, Andrášik R, Bíl M, González‐Suárez M. Roadkill risk and population vulnerability in European birds and mammals. Front Ecol Environ. 2020;18(6):323–8. doi: 10.1002/fee.2216

[25] Wilkins DC, Kockelman KM, Jiang N. Animal-vehicle collisions in Texas: How to protect travelers and animals on roadways. Accid Anal Prev. 2019;131:157–70. doi: 10.1016/j.aap.2019.05.030 31277019

[26] Liu C, Berry PM, Dawson TP, Pearson RG. Selecting thresholds of occurrence in the prediction of species distributions. Ecography. 2005;28(3):385–93. doi: 10.1111/j.0906-7590.2005.03957.x

[27] Chyn K, Lin T-E, Chen Y-K, Chen C-Y, Fitzgerald LA. The magnitude of roadkill in Taiwan: patterns and consequences revealed by citizen science. Biological Conserv. 2019;237:317–26. doi: 10.1016/j.biocon.2019.07.014

[28] Parchizadeh J, Shilling F, Gatta M, Bencini R, Qashqaei AT, Adibi MA, et al. Roads threaten Asiatic cheetahs in Iran. Curr Biol. 2018;28(19):R1141–2. doi: 10.1016/j.cub.2018.09.005 30300597

[29] Moqanaki EM, Cushman SA. All roads lead to Iran: predicting landscape connectivity of the last stronghold for the critically endangered Asiatic cheetah. Anim Conserv. 2016;20(1):29–41. doi: 10.1111/acv.12281

[30] Naderi M, Farashi A, Erdi MA. Persian leopard’s (Panthera pardus saxicolor) unnatural mortality factors analysis in Iran. PLoS One. 2018;13(4):e0195387. doi: 10.1371/journal.pone.0195387 29694391 PMC5918793

[31] Sanei A, Musavi M, Rabiee K, Khosravi MS, Julaee L, Gudarzi F, et al. Distribution, characteristics and conservation of the jungle cat in Iran. IUCN Cat News, 2016;10:51-5.

[32] Akhani H, Djamali M, Ghorbanalizadeh A, Ramezani E. Plant biodiversity of Hyrcanian relict forests, N Iran: an overview of the flora, vegetation, paleoecology and conservation. Pakistan J Botany. 2010;42:231–58.

[33] Sagheb Talebi Kh, Sajedi T, Pourhashemi M. Forests of Iran (A treasure from the past, a hope for the future). Springer Netherlands; 2014.

[34] Moradi S, Sheykhi Ilanloo S, Kafash A, Yousefi M. Identifying high-priority conservation areas for avian biodiversity using species distribution modeling. Ecol Indicat. 2019;97:159–64. doi: 10.1016/j.ecolind.2018.10.003

[35] Yousefi M, Mahmoudi A, Vaissi S, Kafash A. Diversity, diversification and distribution of Iranian vertebrates: the legacy of mountains uplifting, past climatic oscillations, sea level fluctuations and geographical barriers. Biodivers Conserv. 2022;32(1):7–36. doi: 10.1007/s10531-022-02499-2

[36] Mohammadi A, Almasieh K, Clevenger AP, Fatemizadeh F, Rezaei A, Jowkar H, et al. Road expansion: a challenge to conservation of mammals, with particular emphasis on the endangered Asiatic cheetah in Iran. J Nature Conserv. 2018;43:8–18. doi: 10.1016/j.jnc.2018.02.011

[37] Soofi M, Ghoddousi A, Zeppenfeld T, Shokri S, Soufi M, Egli L, et al. Assessing the relationship between illegal hunting of ungulates, wild prey occurrence and livestock depredation rate by large carnivores. J Appl Ecol. 2018;56(2):365–74. doi: 10.1111/1365-2664.13266

[38] Kafash A, Ashrafi S, Yousefi M. Biogeography of bats in Iran: mapping and disentangling environmental and historical drivers of bat richness. J Zool Syst Evol Res. 2021;59(7):1546–56. doi: 10.1111/jzs.12520

[39] Kafash A, Ashrafi S, Yousefi M. Modeling habitat suitability of bats to identify high priority areas for field monitoring and conservation. Environ Sci Pollut Res Int. 2022;29(17):25881–91. doi: 10.1007/s11356-021-17412-7 34851481

[40] Jouladeh-Roudbar A, Ghanavi HR, Doadrio I. Ichthyofauna from Iranian freshwater: annotated checklist, diagnosis, taxonomy, distribution and conservation assessment. Zool Stud. 2020;59:e21. doi: 10.6620/ZS.2020.59-21 33456548 PMC7807176

[41] Yousefi M, Mahmoudi A, Kafash A, Khani A, Kryštufek B. Biogeography of rodents in Iran: species richness, elevational distribution and their environmental correlates. Mammalia. 2022;86(4):309–20. doi: 10.1515/mammalia-2021-0104

[42] Mahmoudi S, Sheykhi Ilanloo S, Keyvanloo Shahrestanaki A, Valizadegan N, Yousefi M. Effect of human-induced forest edges on the understory bird community in Hyrcanian forests in Iran: Implication for conservation and management. Forest Ecol Manag. 2016;382:120–8. doi: 10.1016/j.foreco.2016.10.011

[43] Ashoori A, Kafash A, Varasteh Moradi H, Yousefi M, Kamyab H, Behdarvand N, et al. Habitat modeling of the common pheasant Phasianus colchicus (Galliformes: Phasianidae) in a highly modified landscape: application of species distribution models in the study of a poorly documented bird in Iran. Euro Zoo J. 2018;85(1):372–80. doi: 10.1080/24750263.2018.1510994

[44] Meinecke L, Soofi M, Riechers M, Khorozyan I, Hosseini H, Schwarze S, et al. Crop variety and prey richness affect spatial patterns of human-wildlife conflicts in Iran’s Hyrcanian forests. J Nature Conserv. 2018;43:165–72. doi: 10.1016/j.jnc.2018.04.005

[45] Da Silva LG, Cherem JJ, Kasper CB, Trigo TC, Eizirik E. Mapping wild cat roadkills in southern Brazil: baseline data for species conservation. Catnews. 2014;61:4–7.

[46] Karami M, Ghadirian T, Faizolahi K. The atlas of the mammals of Iran. Tehran: Iran Department of the Environment; 2016.

[47] Masoud Y, Kafash A, Valizadegan N, Ilanloo SS, Rajabizadeh M, Malekoutikhah S, et al. Climate change is a major problem for biodiversity conservation: a systematic review of recent studies in Iran. Contemp Probl Ecol. 2019;12(4):394–403. doi: 10.1134/s1995425519040127

[48] Yousefi M. Challenges and opportunities for biodiversity governance in Iran. Nature Rev Biodiv. 2025. doi: 10.1038/s44358-025-00089-y

[49] Kosarev AN. Physico-geographical conditions of the caspian sea. In: Kostianoy AG, Kosarev AN, editors. The caspian sea environment. Springer; 2005.

[50] Nowell K, Jackson P. Wild Cats. Gland: IUCN; 1996.

[51] Abu-Baker M, Nassar K, Rifai L, Qarqaz M, Al-Melhim W, Amr Z. On the current status and distribution of the Jungle Cat, Felis chaus, in Jordan (Mammalia: Carnivora). Zoo Middle East. 2003;30:5–10.

[52] Duckworth JW, Poole CM, Tizard RJ, Walston JL, Timmins RJ. The Jungle Cat Felis chaus in Indochina: a threatened population of a widespread and adaptable species. Biodivers Conserv. 2005;14(5):1263–80. doi: 10.1007/s10531-004-1653-4

[53] Gray TNE, Timmins RJ, Jathana D, Duckworth JW, Baral H, Mukherjee S. Felis chaus (amended version of 2016 assessment). 2021. 10.2305/IUCN.UK.2021-2.RLTS.T8540A200639312

[54] Mousavi M, Rabiee K, Khosravi MS, Julaee L, Gudarzi F, Jaafari B, et al. Distribution, characteristics and conservation of the jungle cat in Iran. Catnews. 2016;10:51–5.

[55] Ziaie H. A field guide to the mammals of Iran. Tehran: Iranian Wildlife Center; 2008.

[56] Fick SE, Hijmans RJ. WorldClim 2: new 1‐km spatial resolution climate surfaces for global land areas. Intl J Climatol. 2017;37(12):4302–15. doi: 10.1002/joc.5086

[57] Venter O, Sanderson EW, Magrach A, Allan JR, Beher J, Jones KR, et al. Sixteen years of change in the global terrestrial human footprint and implications for biodiversity conservation. Nat Commun. 2016;7:12558. doi: 10.1038/ncomms12558 27552116 PMC4996975

[58] Venter O, Sanderson EW, Magrach A, Allan JR, Beher J, Jones KR, et al. Global terrestrial Human Footprint maps for 1993 and 2009. Sci Data. 2016;3:160067. doi: 10.1038/sdata.2016.67 27552448 PMC5127486

[59] Jarvis A, Reuter HI, Nelson A, Guevara E. Hole-filled SRTM for the globe Version 4. CGIAR-CSI SRTM 90m Database; 2008. https://srtm.csi.cgiar.org/

[60] Quinn GP, Keough MJ. Experimental designs and data analysis for biologists. Cambridge: Cambridge University Press; 2002.

[61] Naimi B. Uncertainty analysis for species distribution models. 2015.

[62] R Core Team. R: a language and environment for statistical computing. Vienna: R Foundation for Statistical Computing; 2025.

[63] Guisan A, Thuiller W, Zimmermann NE. Habitat suitability and distribution models: with applications in R. Cambridge: Cambridge University Press; 2017.

[64] Merow C, Smith MJ, Silander JA Jr. A practical guide to MaxEnt for modeling species’ distributions: what it does, and why inputs and settings matter. Ecography. 2013;36(10):1058–69. doi: 10.1111/j.1600-0587.2013.07872.x

[65] Yousefi M, Heydari-Guran S, Kafash A, Ghasidian E. Species distribution models advance our knowledge of the Neanderthals’ paleoecology on the Iranian Plateau. Sci Rep. 2020;10(1):14248. doi: 10.1038/s41598-020-71166-9 32859969 PMC7455561

[66] Asgharzadeh M, Alesheikh AA, Yousefi M. Disentangling the impacts of climate and land cover changes on habitat suitability of common pheasant Phasianus colchicus along elevational gradients in Iran. Environ Sci Pollut Res Int. 2023;30(21):60958–66. doi: 10.1007/s11356-023-26742-7 37042917

[67] Gholamhosseini A, Yousefi M, Esmaeili HR. Predicting climate change impacts on the distribution of endemic fish Cyprinion muscatense in the Arabian Peninsula. Ecol Evol. 2024;14(7):e11720. doi: 10.1002/ece3.11720 38988343 PMC11236460

[68] Carné A, Vieites DR, Sillero N. Potential effects of climate change on the threatened Malagasy poison frogs: a multispecies approach. Ecosphere. 2025;16(6). doi: 10.1002/ecs2.70315

[69] Kafash A, Bojd AAH, Pintor A, Grünig M, Yousefi M, Hassanpour G. Applying ensemble ecological niche modeling to identify high risk areas for scorpions’ sting. Ecol Evol. 2025;15(7):e71713. doi: 10.1002/ece3.71713 40625335 PMC12231216

[70] Pinkert S, Farwig N, Kawahara AY, Jetz W. Global hotspots of butterfly diversity are threatened in a warming world. Nat Ecol Evol. 2025;9(5):789–800. doi: 10.1038/s41559-025-02664-0 40128452 PMC12066357

[71] Phillips SJ, Anderson RP, Schapire RE. Maximum entropy modeling of species geographic distributions. Ecological Modelling. 2006;190(3–4):231–59. doi: 10.1016/j.ecolmodel.2005.03.026

[72] Phillips SJ, Dudík M. Modeling of species distributions with Maxent: new extensions and a comprehensive evaluation. Ecography. 2008;0(0):080328142746259-???. doi: 10.1111/j.0906-7590.2007.5203.x

[73] Cobos ME, Peterson AT, Barve N, Osorio-Olvera L. kuenm: an R package for detailed development of ecological niche models using Maxent. PeerJ. 2019;7:e6281. doi: 10.7717/peerj.6281 PMC6368831 30755826

[74] Heumann BW, Walsh SJ, McDaniel PM. Assessing the application of a geographic presence-only model for land suitability mapping. Ecol Inform. 2011;6(5):257–69. doi: 10.1016/j.ecoinf.2011.04.004 21860606 PMC3158664

[75] Kafash A, Malakoutikhah S, Yousefi M, Ataei F, Heidari H, Rastegar-Pouyani E. The gray toad-headed agama, phrynocephalus scutellatus, on the iranian plateau: the degree of niche overlap depends on the phylogenetic distance. Zoology in the Middle East. 2017;64(1):47–54. doi: 10.1080/09397140.2017.1401309

[76] Swets JA. Measuring the accuracy of diagnostic systems. Science. 1988;240(4857):1285–93. doi: 10.1126/science.3287615 3287615

[77] Yousefi M, Kafash A, Malakoutikhah S, Ashoori A, Khani A, Mehdizade Y, et al. Distance to international border shapes the distribution pattern of the growing Little BustardTetrax tetraxwinter population in Northern Iran. Bird Conserv Inter. 2017;28(4):499–508. doi: 10.1017/s0959270917000181

[78] Burnham KP, Anderson DR. Model selection and multimodel inference: a practical information-theoretic approach. New York, USA: Springer; 2002.

[79] Bartón K. MuMIn: Multi-model inference. 2025.

[80] Phillips SJ, Dudík M, Schapire RE. Maxent software for modeling species niches and distributions. Accessed 2019 June 4. http://biodiversityinformatics.amnh.org/open_source/maxent/

[81] Darvishsefat A. Atlas of protected areas of Iran. Tehran: Department of Environment of Iran; 2008.

[82] Meza-Joya FL, Ramos E, Cardona D. Spatio-temporal patterns of mammal road mortality in middle magdalena valley, Colombia. Oecol Aust. 2019;23(03):575–88. doi: 10.4257/oeco.2019.2303.15

[83] Miranda JES, de Melo FR, Umetsu RK. Are roadkill hotspots in the Cerrado equal among groups of vertebrates?. Environ Manag. 2020;65:565–73. 10.1007/s00267-020-01263-y 32060629

[84] Mousavi SM, Rezaei HR, Naderi S. Prediction of potential distribution of wild cat Felis silvestris using maximum entropy. Appl Ecol. 2018;10:19–24.

[85] Zimmermann Teixeira F, Kindel A, Hartz SM, Mitchell S, Fahrig L. When road‐kill hotspots do not indicate the best sites for road‐kill mitigation. J Appl Ecol. 2017;54(5):1544–51. doi: 10.1111/1365-2664.12870

[86] Kazemi V, Jafari H, Yavari A. Spatio-temporal patterns of wildlife road mortality in golestan national park-North East of Iran. Open Journal of Ecology. 2016;6:312–24.

[87] Mukherjee S, Groves C. Geographic variation in jungle cat (Felis chaus Schreber, 1777) (Mammalia, Carnivora, Felidae) body size: is competition responsible?. Biol J Linnean Soc. 2007;92(1):163–72. doi: 10.1111/j.1095-8312.2007.00838.x

[88] Shahidinejad F, Danehkar A, Alizadeh Shabani A, Sobhani P. Investigation of road accidents on the loss of wildlife species in the different provinces of Iran. J Natural Environ. 2024;77:199–211.

[89] Moqanaki E, Farhadinia MS, Mousavi M, Breitenmoser U. Distribution and conservation status of the Eurasian lynx in Iran: a preliminary assessment. Cat News. 2010;53:32–5.

